# Annulus fibrosus cell sheets limit disc degeneration in a rat annulus fibrosus injury model

**DOI:** 10.1002/jsp2.1050

**Published:** 2019-06-17

**Authors:** Tadashi Nukaga, Daisuke Sakai, Jordy Schol, Masato Sato, Masahiko Watanabe

**Affiliations:** ^1^ Department of Orthopaedic Surgery Tokai University School of Medicine Isehara Kanagawa Japan

**Keywords:** annulus fibrosis, cell sheet, cell transplantation, disc herniation, intervertebral disc, rat, regeneration, tissue engineering

## Abstract

In recent years, studies have explored novel approaches for cell transplantation to enable annulus fibrosus (AF) regeneration of the intervertebral disc in particular for lumbar disc herniation. Nevertheless, successful engraftment of cells is structurally challenging, and no definitive method has yet been established. This study investigated the potential of cell sheet technology to facilitate cell engraftment for AF repair. AF injury was induced by a 1 × 1 mm defect in rat tails after which AF cell sheets were transplanted. Its regenerative effects were compared to a nondegenerated and degeneration only conditions. Degenerative changes of the entire intervertebral disc were examined by disc height measurements, histology, and immunohistochemistry for 4‐, 8‐, and 12‐weeks post‐transplantation. Cell engraftment was confirmed by tracing PKH26 fluorescent dyed AF cells. In the transplant group, disc degeneration was significantly suppressed after 4, 8, and 12 weeks when compared with the degenerative group, as indicated by histological scoring and DHI observations. At 2 and 4 weeks after transplant, PKH26 positive cells could be detected in defect region and surrounding AF. The results suggest cell engraftment into AF tissue could be established by the cell sheet technology without additional scaffolding or adhesives. In short, AF cell sheets appear to be an effective and accessible tool for AF repair and to support intervertebral disc regeneration.

## 1 INTRODUCTION

On an annual basis 1% of the United States adult population will be afflicted by lumbar disc herniation, affecting an estimated 2.8 million people each year.^1^ Contemporary treatment options involve physical therapy, corticosteroid injection, and nonsteroidal anti‐inflammatory drug administration, which is able to alleviate symptoms for a large fraction of patients.^2^ Alternatively, herniated disc material can be excised to alleviate inflammation and potentially decompress neighboring nerves. As such, 300 000 discectomies are predicted to be performed.^3^ Nevertheless, discectomy of herniated intervertebral discs (IVD**)** presents a recurrence rate between 3.5% and 12%,^4^, ^5^ with reporting as high as 23% when including asymptomatic cases.^6^ Contemporary treatments do not involve repair of the annulus fibrosus (AF), which is likely to be the main cause of herniation recurrence. Moreover, untreated herniated AF may result in chronic back pain,^7^ by a process involving the invasion of sensory nerves into the IVD via the defected AF area,^8^ and an increase in inflammatory cytokines.^9^

For these reasons, a multitude of studies has explored different approaches to establish AF repair.^10^, ^11^, ^12^, ^13^ One approach that is showing potential for IVD repair, is cell transplantation, with initial clinical trials aimed at repairing the IVD via the nucleus pulposus (NP) showing promising safety and efficacy outcomes.^14^ Nevertheless, cell transplantation has been shown challenging for the AF, in particular, due to the structural difficulty of attaching cells to this tissue. Unlike the NP, which is enclosed by the AF and vertebrae, allowing for easy cell retention, the AF has no structural components encasing the AF and a potential transplant. Scaffolds are thus likely required in order to keep transplanted cells localized. A variety of studies^15^, ^16^ have reported on AF cell transplantation by applying adhering scaffolds, however realization of cell engraftment remained limited. Moreover, the AF is a layered tissue structure that gradually changes morphology and composition from the inner to outer the layers, thus likely requiring specialized tissue engineering approaches for successful long‐term repair.^12^

A promising new technology in which cell sheets are produced has been used for regenerating various organs.^17^, ^18^, ^19^, ^20^, ^21^ Instead of obtaining single or aggregated cells in suspension, cell sheet products are comprised of a layer of monolayer cultured cells.^19^, ^22^ The cell sheets can be obtained by, for example, culturing specific cell populations on a thermoresponsive polymer, which can thereafter be detached from the culture plate by altering the environmental temperature, giving rise to a dissociated but intact sheet of the cultured cells.^22^ The rate of cell engraftment has shown to improve using cell sheets rather than single cells^17^ and has successfully been applied for a variety of organs, such as the heart,^17^ cornea,^18^ cartilage,^19^ spinal cord,^20^ and bone.^21^ In this proof‐of‐concept study, we aimed to investigate whether this cell sheet technology could be applied as an effective method for enabling AF defect repair and limit consequential IVD degeneration. Moreover, we wanted to investigate if cell sheets allowed for implant attachment and subsequent cell engraftment into the AF.

## 2 MATERIALS AND METHODS

### 2.1 AF cell sheet preparation

**Figure 1:**
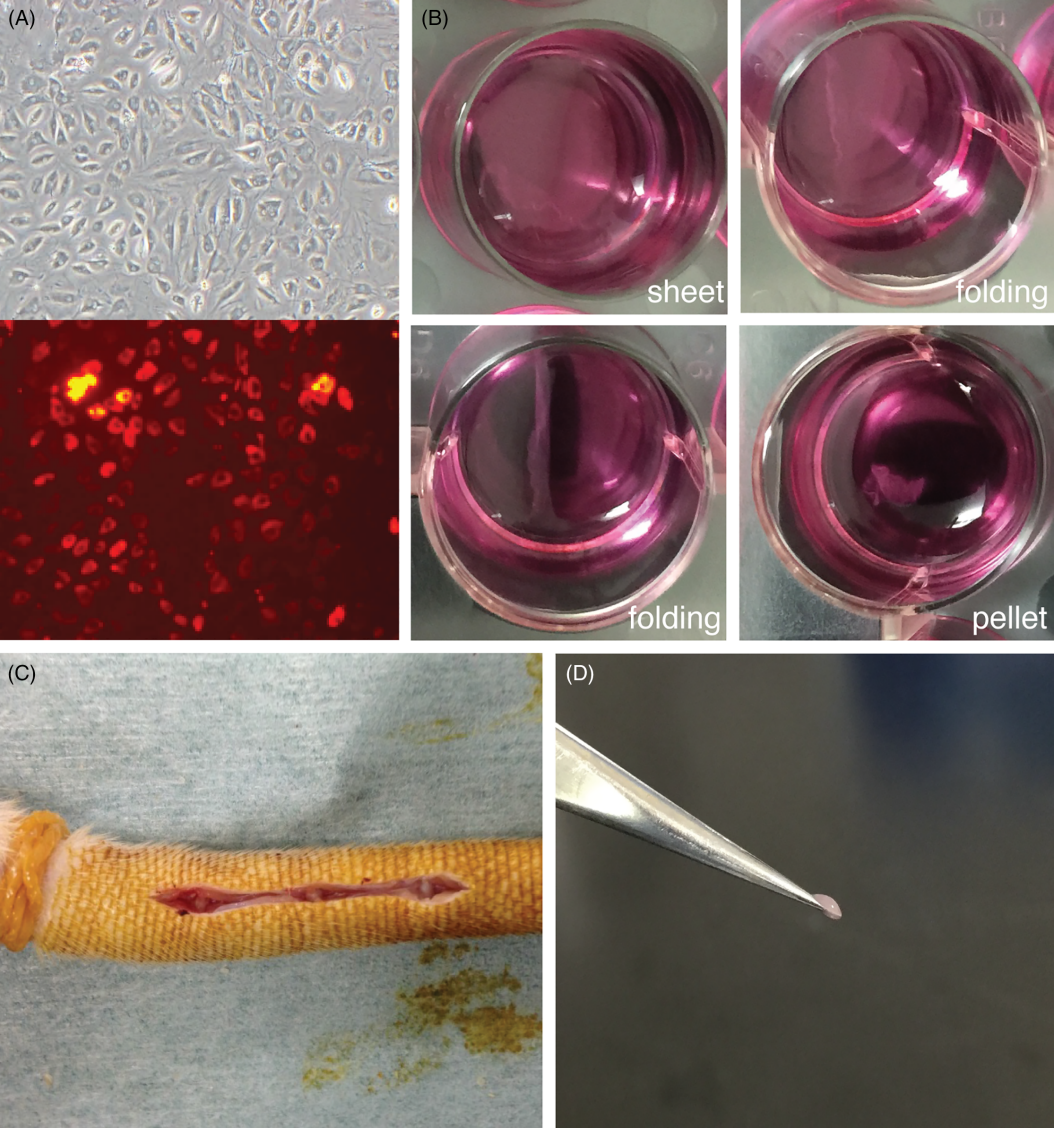
Surgical annulus fibrosus defects created in rat tail. A, Representative pictures taken of annulus fibrosus cells stained with PKH26, just prior to seeding on cell sheet cultureware. B, Representative overview of temporal monolayer detachment when subjected to room temperature. Culture of the annulus fibrosus cells on thermoresponsive polymer forms a typical cell sheet, which when subjected to room temperature starts to detach, fold, and thereafter forming a pellet, respectively. C, Surgery was performed after avascularizition of rat's tail by applying rubber bands at the tail base. Subsequently, Cy 7/8, 8/9, and 9/10 intervertebral discs were exposed in each animal. A 1 × 1 mm defect was created implementing a microscope. D, Picture of AF cell sheet pellet held by forceps just prior to transplantation

This study and all procedures were conducted in accordance with protocols approved by the Tokai University, School of Medicine committee for safe animal experimentation (No. 164018). AF cells were obtained from the caudal vertebra of five, 11‐week‐old female Sprague Dawley rats (CLEA Japan Inc., Tokyo, Japan) according to previous work of Nakai et al.^23^ The collected AF tissues were treated with 1X TrypLE Express (Gibco, Grand Island, New York) for 30 minutes, followed by treatment with 0.25 mg/mL Liberase DL (Roche, Basel, Switzerland) for 60 minutes in a temperature‐controlled room at 37°C. The cells obtained from a single rat were then seeded onto a single 6‐well plates well (Becton Dickinson, Franklin Lakes, New Jersey) according to previously published work,^23^ and were cultured in Dulbecco's modified Eagle's medium (Gibco), supplemented with 10% fetal bovine serum (Sigma‐Aldrich, St. Louis, Missouri), 100 U/mL penicillin (Gibco), and 100 mg/mL streptomycin (Gibco), for 1 week at 2% O_2_. Cultured AF cells were seeded onto 24‐well UpCell cultureware (CellSeed Inc., Tokyo, Japan) at 1 × 10^4^ cells/cm^2^ and were cultured under identical conditions for an additional week. At the time of transplantation, the UpCell cultureware was removed from the incubator and allowed to stand for 30 minutes at room temperature. (Figure 1A) The cell sheets were then collected using forceps (Figure 1C).

### 2.2 Labeling AF cells using PKH26

In order to confirm successful engraftment, a portion of the AF cell was labeled with PKH26 (Sigma‐Aldrich). After 1‐week monolayer culture in 6‐well plates, the AF cells were labeled according to the manufacturer's instructions (Sigma‐Aldrich) followed by passaging them onto separate UpCell cultureware plates. In total, two rats received the cell sheets comprised of PKH26 labeled AF cells in 3 IVDs each.

### 2.3 Rat tail AF injury model and cell sheet transplantation

A total of 27, 11‐week‐old, female Sprague Dawley rats were applied for this study. The rats were divided into three groups of nine rats as follows: healthy sham control (group C), degenerative group (group D), and sheet group (group S). Group C was defined as those without AF damage, group D was defined as those only subjected to AF damage, and group S was defined as those who underwent surgical cell sheets transplantation after induced AF damage. Finally, within each group, the rats were assigned to 4, 8, and 12 weeks postoperative time points of sacrifice (each; n = 9). A rat tail AF injury model was created according to methods of Borde et al. and Kazezian et al. with minor modifications.^24^, ^25^ Surgery was performed under general anesthesia using 2.5% isoflurane inhalation while in a prone position. The base of the tail was avascularized using rubber bands. The tail IVDs Cy 7/8, 8/9, and 9/10 were exposed in each animal. A 1 × 1 mm defect approximately 0.5 mm deep (without releasing NP tissue) generated using a microscope for group S and D (Figure 1B). Subsequently, for group S, two layers of AF cell sheets were directly applied as pellets into the AF defects. (Figure 1C) Furthermore, two rats of group S received PKH26 labeled cell sheet as the transplant product and were assigned to be sacrificed at week 2 and 4 post‐transplantation. At week 4, 8, and 12 corresponding rats were sacrificed under continuous 2.5% isoflurane inhalation by excessive cardiac pentobarbital injection.

### 2.4 Gross anatomy

IVDs obtained from 27 rats (81 discs) were fixed in 4% paraformaldehyde and decalcified using Decalcifying Solution A (Wako, Osaka, Japan) for 7 days. The samples were dissected at the center of the vertical plane to ensure that the disc center could be accessed for macroscopic evaluation. In a blinded fashion Thompson grading system^26^ was used to assess disc degeneration on macroscopic levels, by two investigator.

### 2.5 Histological examination

Each IVD was fixed and decalcified and were thereafter embedded in paraffin. Paraffin blocks were sectioned and stained using hematoxylin/eosin (H&E) and 1 g/L Safranin‐O (Merck, Kenilworth, New Jersey, USA) 800 mg/L Fast Green FCF staining (Merck) solution. Microscopic images were captured using the BZ‐9000 Biorevo fluorescence microscope (Keyence, Osaka, Japan). The degree of disc degeneration was evaluated from H&E stained sections applying the grading system of Nishimura and Mochida.^27^ The grading system of Nomura was used to evaluate the degree of NP degeneration.^28^ Two involved researchers (T. N. and J. S.) scored the samples in a blinded fashion.

### 2.6 Disc height index assessment

Disc height was measured applying H&E stained sections according to the method of Nishimura and Mochida.^27^ In short, from HE‐sections of the center of the IVD, the area between cephalic and caudal endplates was calculated and divided by the average width. Relative DHI was defined as the ratio of DHI compared to the mean DHI of group C, at week 4.

### 2.7 Immunohistochemistry

Immunohistochemical staining was performed following standard protocol using collagen Iα antibody (1:100, NBP1‐30054, Novus Biologicals, Littleton, Colorado) overnight at 4°C, rat anti‐rabbit Ig horseradish peroxidase‐conjugated antibody (Histofine SimpleStain, Nichirei BioSciences, Tokyo, Japan) for 1 hour at room temperature and approximately 3 minutes incubation in 30 mg/ 3,3′‐diaminobenzidine tetrahydrochloride 150 mL 0.05 M Tris‐HCl (7.0)/25 μL H_2_O_2_.

### 2.8 Engraftment of transplanted cells

PKH26 staining was determined at 91.4% ± 3.2% prior to seeding on cultureware. Paraffin section obtained from IVD tissue treated with PKH26‐labeled cells was evaluated under the TRITC filter, BZ‐9000 Biorevo fluorescence microscope (Keyence). Pictures were taken in the region of the AF at the dorsal side. The number of PKH26‐positive cells per 1 mm^2^ was manually counted.

### 2.9 Statistical analysis

The analysis of variance test was used to analyze the disc height, gross anatomical, and histological examinations. Fisher's test was used for posthoc testing. The results are presented as mean ± SD, and a *P* value <.05 was deemed significantly different.

## 3 RESULTS

### 3.1 Disc height index assessment

**Figure 2:**
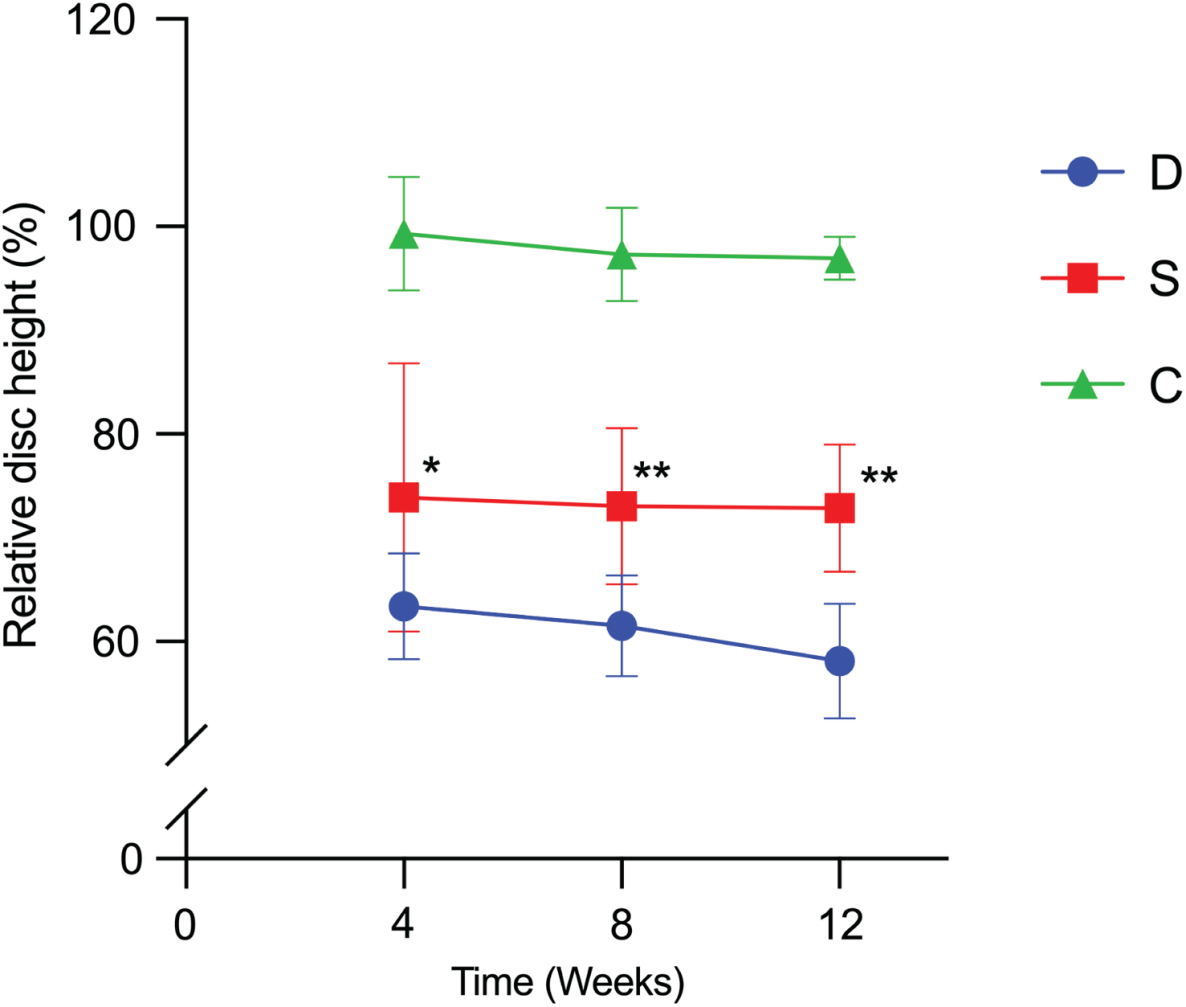
Disc height index assessment. In group D, disc height index (DHI) decreased to 63% 4 weeks after injury induction, and showed further DHI deterioration to 58% at week 12. In contrast, DHI in group S could be maintained at 72% after 4 weeks, and was significantly higher in group S compared to group D at 4, 8, and 12 weeks. Abbreviations: D: AF defect only group, S: AF defect treated with sheet transplantation group, C: sham group * *P* <.05, ** *P* <.01 comparing group S to group D (n = 9)

In group D, the disc height decreased to 63% at 4 weeks after injury and further showed a decreasing trend to 58% at week 12. The DHI in group C was maintained at approximately 100% throughout the experimental period. In contrast, DHI of group S was measured at 72% at the 4 weeks timepoint, which could approximately be maintained throughout the experiments. DHI measured for group S demonstrated significant improvement compared to group D at week 4 (*P* <.05), and at weeks 8 and 12 (*P* <.01) (Figure 2).

### 3.2 Gross anatomy (Thompson grading)

**Figure 3:**
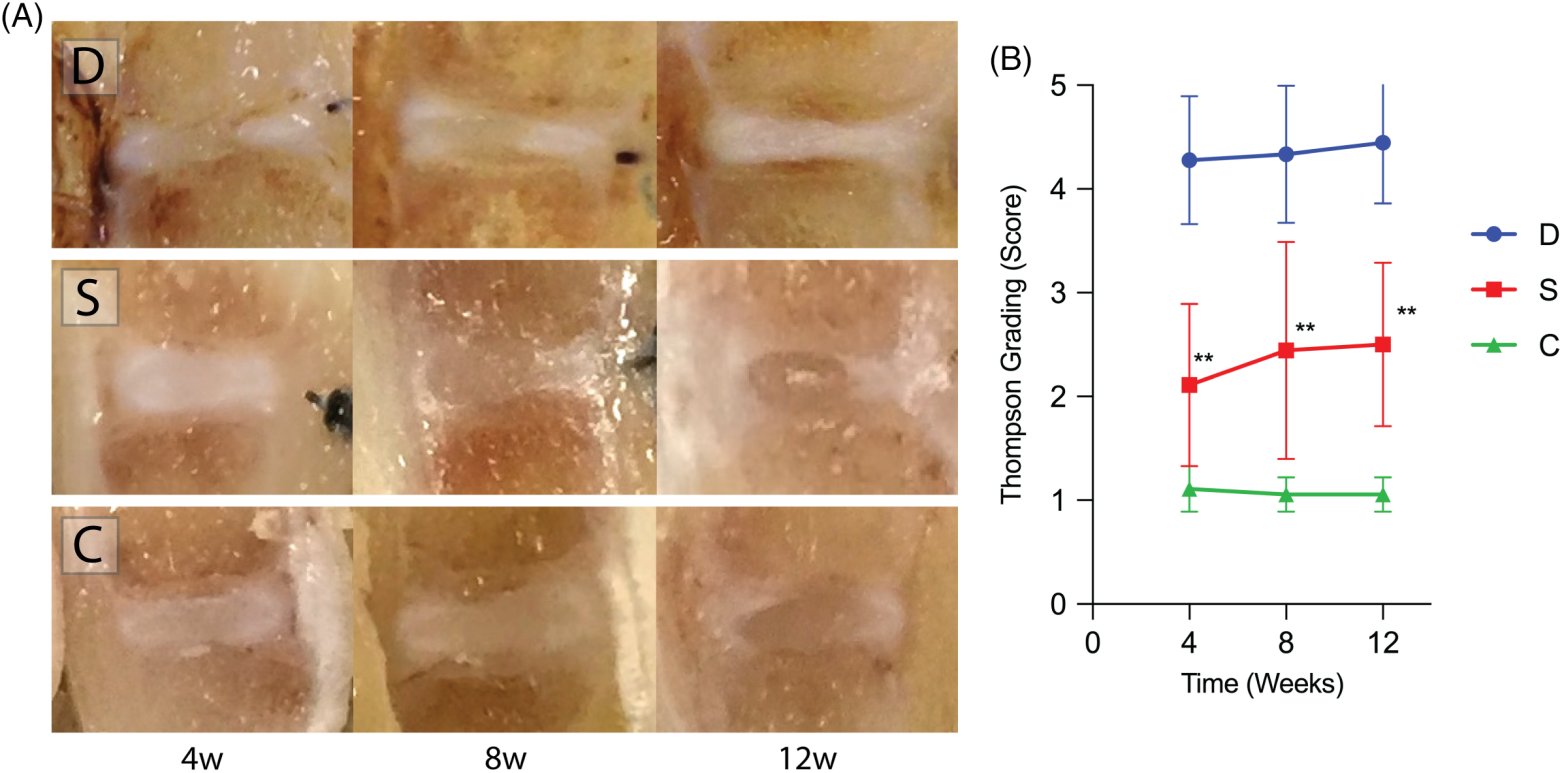
Gross anatomical change observations. A, Macroscopic observations revealed a decrease in disc height and disappearance of NP structure in group D. Disc height was maintained in group S, and NP structure was similar to that of group C. B, Thompson grading system score was 4.1 at week 4 in group D but gradually degenerated to 4.4 after 12 weeks. In contrast, the score was 2.5 at week 12 in group S. Thompson grading was significantly enhanced at weeks 4, 8, and 12 in group S compared to that in group D. Abbreviations: D: AF defect only group, S: AF defect treated with sheet transplantation group, C: sham group ** *P* <.01, comparing group S to group D (n = 9)

From the macroscopic images, a clear differentiation between gelatinous NP and fibrous AF could be made for group S and C samples, however, NP‐AF distinction was lost for group D specimen (Figure 3A). Applying the Thompson grading system score resulted in a score of 4.1 at week 4 for group D, which gradually deteriorated to a score of 4.4 at 12 weeks. In contrast, in group S Thompson score peaked at 2.5 on week 12. Thompson scores were significantly improved (*P* <.01) at weeks 4, 8, and 12 in group S compared to group D (Figure 3B).

### 3.3 Histology and histological scoring

**Figure 4:**
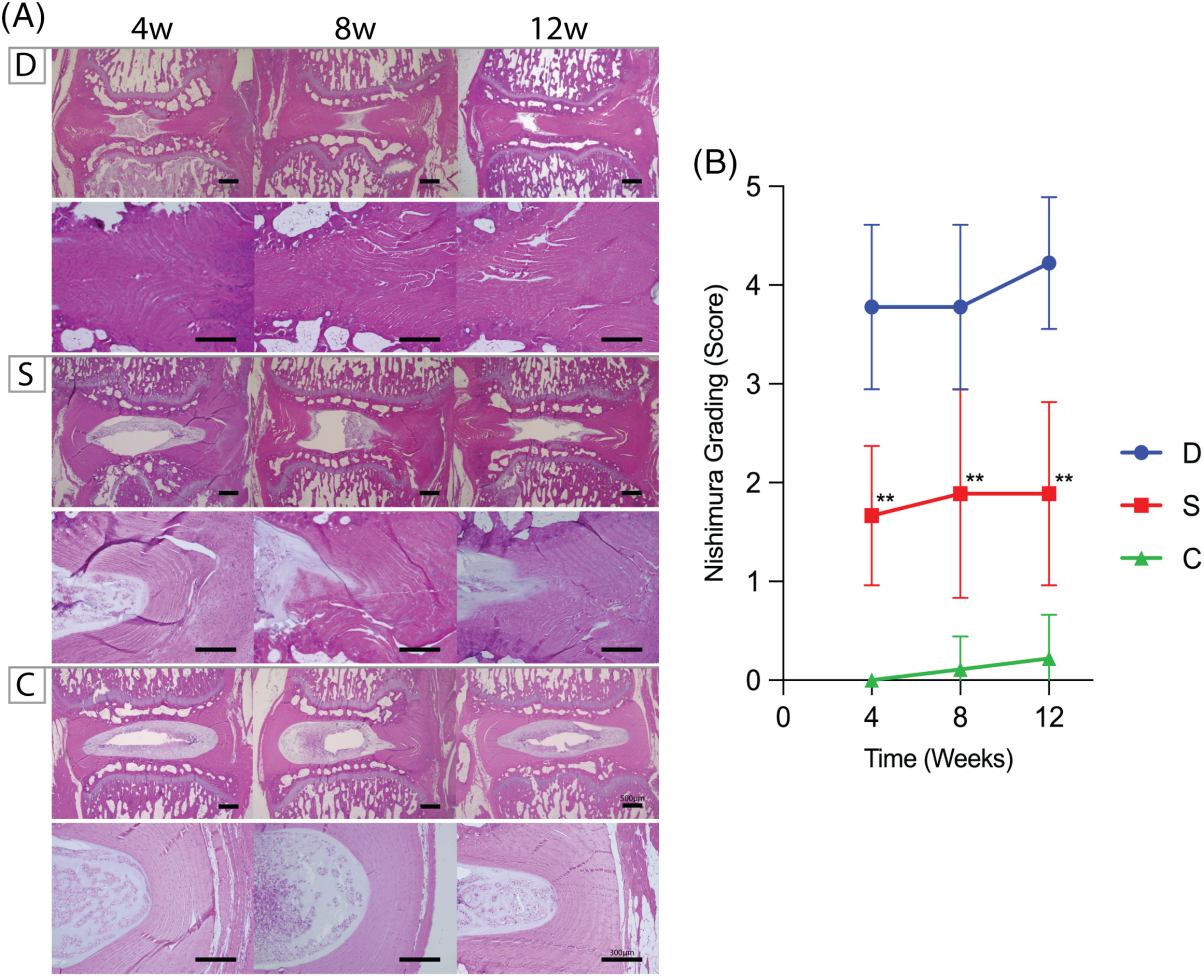
Hematoxylin/eosin staining for assessment of annulus fibrosus structural changes. A, The inner AF structure was inverted in group D, and evident degeneration could be observed. In group S, AF structure resembled that of group C, indicating regeneration of the defected area (scale bar equates 500 and 300 μm for IVD overview and AF focused pictures, respectively). B, Applying the Nishimura grading system resulted in scores of 3.7 at week 4 in group D but further declined to 4.2 at week 12. In contrast, the score for group S was 1.88 at week 12. According to Nishimura grading, degeneration was significantly halted at weeks 4, 8, and 12 in group S compared to that in group D. Abbreviations: D: AF defect only group, S: AF defect treated with sheet transplantation group, C: sham group ** *P* <.01, comparing group S to group D (n = 9)

**Figure 5:**
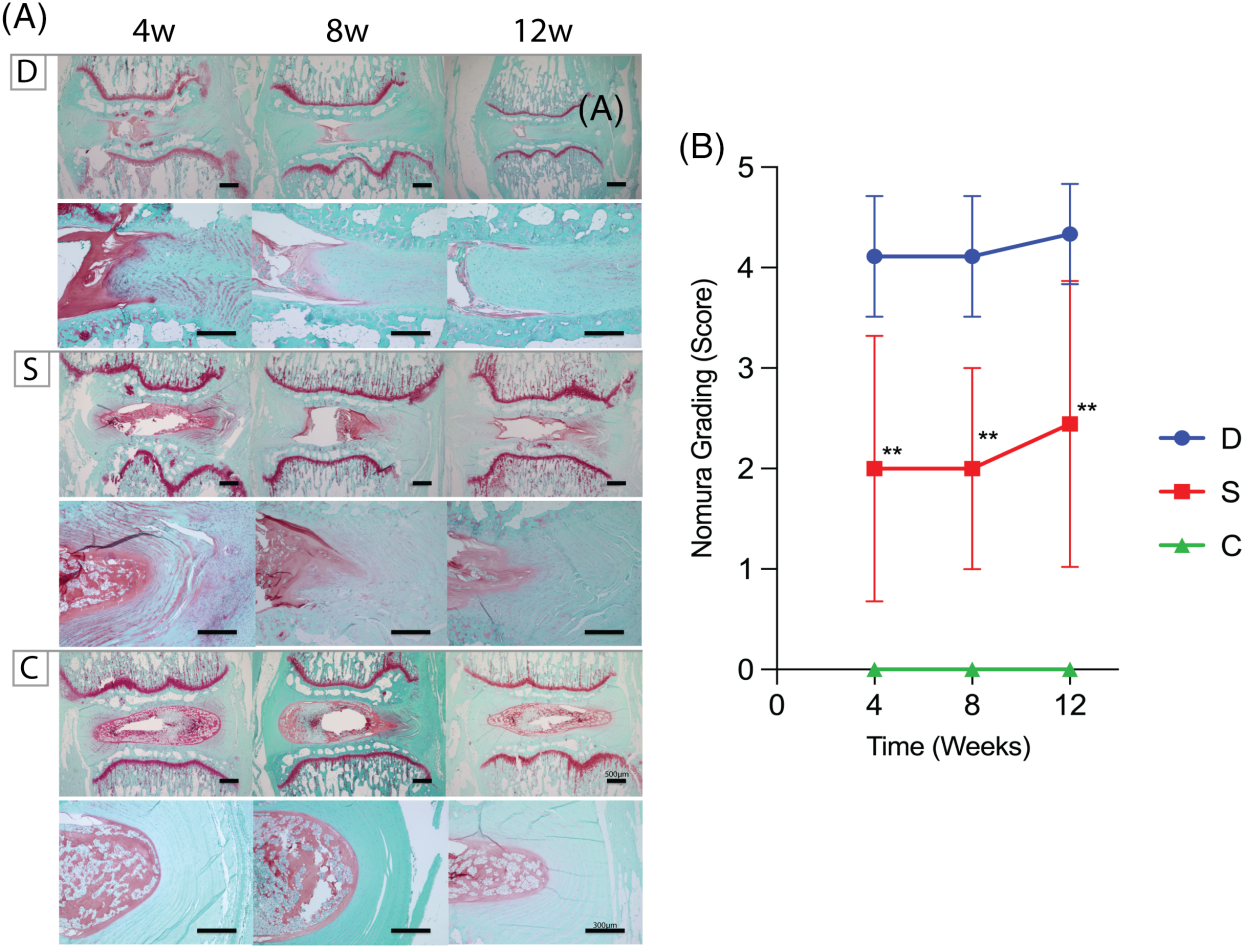
Safranin‐O/fast green staining for nucleus pulposus assessment. A, In group D, the degree of Safranin‐O (red) staining decreased in the nucleus pulposus (NP) tissue, which was replaced by invading annulus fibrosus (AF) or fibrotic connective tissue. Contrary, the NP structure in group S showed maintenance of proteoglycan content as indicated by Safranin‐O staining at similar levels to group C (scale bar equates 500 and 300 μm for IVD overview and AF focused pictures, respectively). B, The Nomura grading system resulted in scores of 4.1 at week 4 in group D and further deteriorated to 4.3 at week 12. Conversely, the score was 2.4 at week 12 for group S. Degeneration was significantly arrested at weeks 4, 8, and 12 in group S compared to that in group D. ** *P* <.01, comparing group S to group D. Abbreviations: D: AF defect only group, S: AF defect treated with sheet transplantation group, C: sham group (n = 9)

The suppressive effect of cell transplantation on degeneration was further confirmed by histological evaluations. H&E staining showed the disappearance of a typical NP structure in group D, a marked inversion of the AF inner layers, and a decrease in disc height. In group S, the NP structure, AF inner layer, and disc height were better preserved than group D (Figure 4B). The Nishimura grading system score was 3.7 at week 4 in group D, but further declined to 4.2 at week 12. In contrast, a maximum score of 1.88 for group S was observed at week 12. Degeneration was significantly arrested (*P* <.01) at weeks 4, 8, and 12 in group S compared to group D (Figure 4B). Safranin‐O/Fast green staining further confirmed NP degeneration progressing from week 4 in group D, as observed by the decrease in Safranin‐O staining and intensity. Instead, AF or connective tissue invaded the degenerating NP for group D. Contrarily, the NP in groups S and C both showed the ability to retain their proteoglycan content as indicated by the preservation of Safranin‐O intensity (Figure 5A). Quantification of degeneration by the Nomura grading system score showed a score of 4.1 at week 4 in group D and declined to 4.3 at week 12. Conversely, the score was 2.4 at week 12 in group S, resulting in a significant improvement (*P* <.01) at weeks 4, 8, and 12 in group S compared to that in group D (Figure 5B).

### 3.4 Immunohistochemistry staining

**Figure 6:**
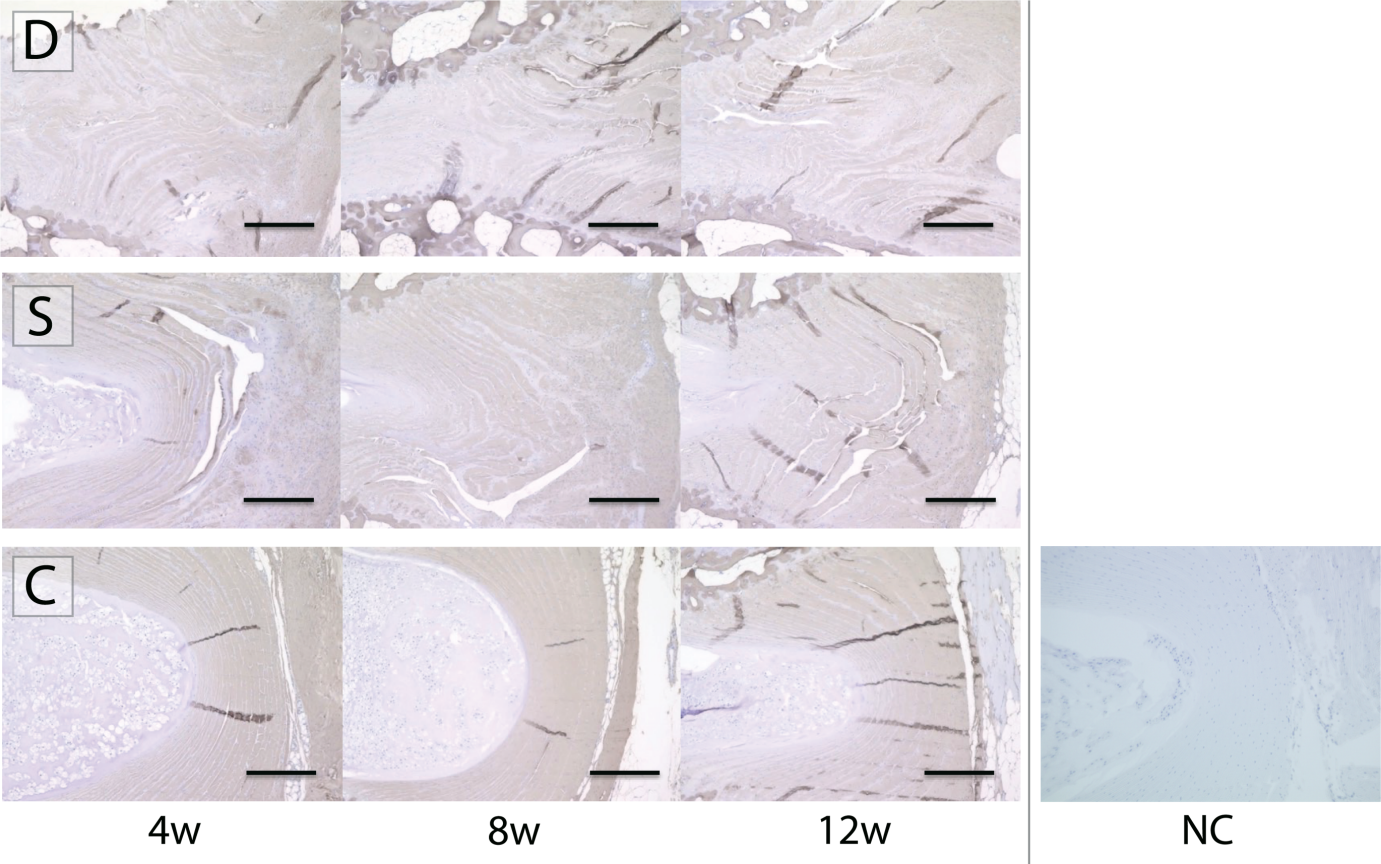
Immunohistochemistry staining for type I collagen. Immunohistochemical staining targeting type I collagen revealed severe disorganization of the annulus fibrosus (AF), and a decrease in staining intensity for group D. On the contrary, the arc‐shaped and layered AF structure with immunoreactivity for type I collagen staining were mostly maintained in group S when compared to group C. Presented images show the AF at site of surgery, defect, and transplant. Abbreviations: D: AF defect only group, S: AF defect treated with sheet transplantation group, C: sham group, NC: negative control of sample stained without primary antibody (scale bar = 300 μm)

The arc‐shaped and layered composition of the AF was disordered, and the degree of type I collagen staining was reduced in group D; however, these were maintained both in groups S and C (Figure 6).

### 3.5 Survival and migration of transplanted AF cells

**Figure 7:**
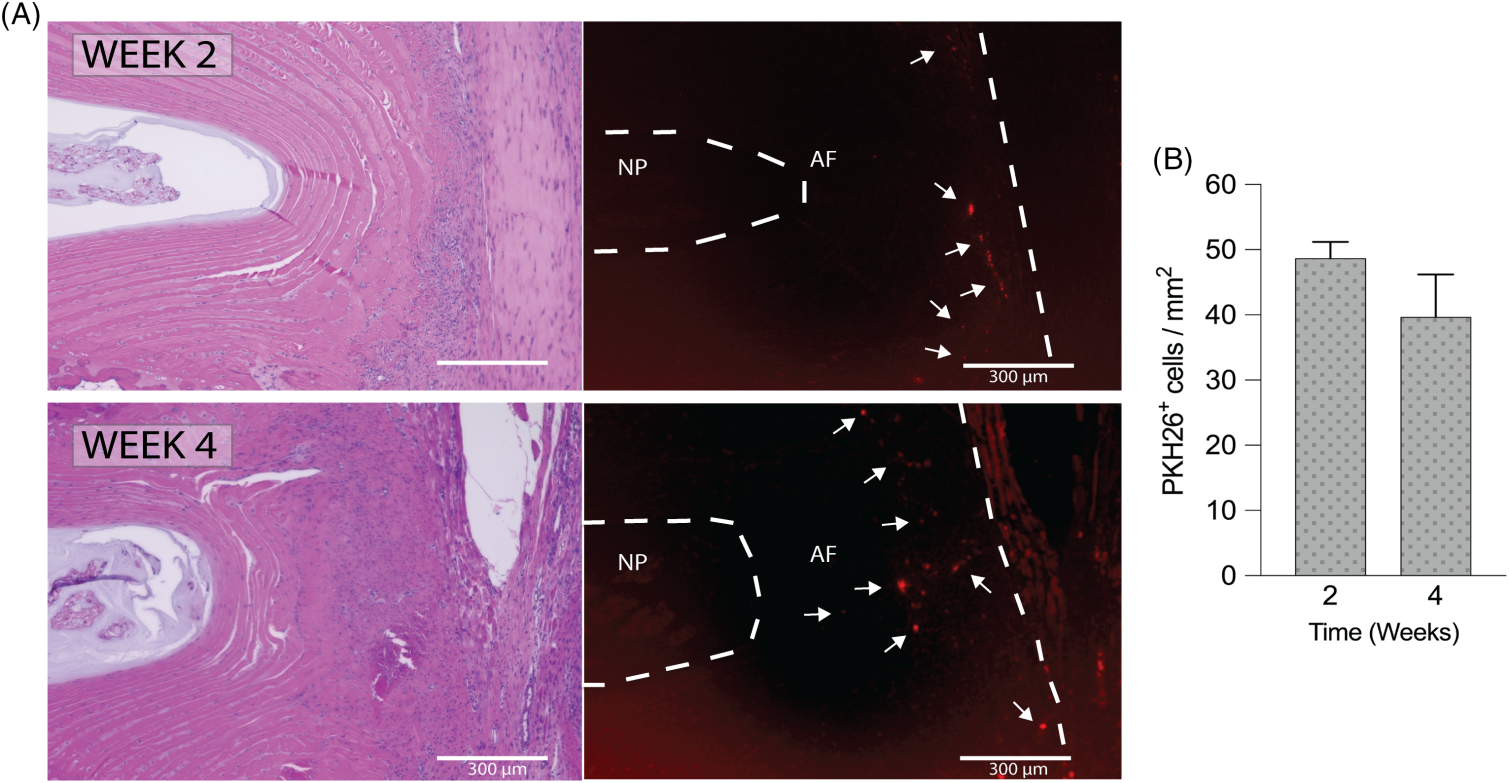
PKH26‐labeling reveals transplanted cell migration and survival in annulus fibrosus. A, A number of transplanted PKH26‐labeled (red) cells were observed in the annulus fibrosus area at 2 and 4 weeks after transplantation. White arrows indicate PKH26 positive cell clusters (scale bar = 300 μm). B, PKH26 positive cell count revealed 48.6 ± 2.0 cells/mm^2^ at 2 weeks post‐transplantation and 39.7 ± 5.3 cells/mm^2^ at 4 weeks post‐transplantation (n = 3)

PKH26‐labeled cells could be detected in the AF in close proximity to the defected area at both 2 and 4 weeks after transplantation (Figure 7A). Quantification of the number of cells showed an average of 48.6 ± 2.0 cells/mm^2^ 2 weeks post‐transplantation and 39.7 ± 5.3 cells/mm^2^ 4 weeks post‐transplantation (Figure 7B).

## 4 DISCUSSION

The ability to repair AF tissue is limited, and a simple mechanical suture remains insufficient.^28^ Implants, such as Inclose or Xclose (ANULEX Technology, Minnetonka, Minnesota), have been developed, but are no longer available as they have caused several complications^29^ and clinical trials have suggested no significant benefits with regard to prevention of reherniation.^30^ At current, Barricaid (Intrinsic Therapeutics, Woburn, Massachusetts) is the only commercially available implant,^29^ for which a significant decrease in herniation recurrence risk could be observed up to 2 years after surgery.^31^ Nevertheless, Barricaid`s long‐term effects remain to be studied, in particular as Barricaid operates by bone anchors and because no in situ AF regeneration was evaluated. Investigations on biomaterial development for AF repair are ongoing and are at various stages of development. A variety of hydrogels designs have been assessed and generally show promise particularly emphasizing biomechanical restoration.^32^ As exemplified by the work of Likhitpanichkul et al, which demonstrated that a fibrin‐genipin composition applied as a sealant for a large AF defect in bovine IVD within a bioreactor culture system, could limit the biomechanical deterioration resulting from the defect.^33^ The observation period, however, was relatively short and although cell infiltration could be observed, it remains to be determined whether application of cell engraftment is required, especially considering the limited densities and potency of endemic IVD cells.^34^, ^35^, ^36^

Cell migration is observed more frequently in AF compared to NP,^37^, ^38^ nevertheless AF's regenerative ability remains rather limited.^39^ AF is a type of tissue that comprises distinguishable and intricately designed layers, which gradually change from the inner to outer AF.^40^ High technological skills, sophisticated biomaterials, and tissue engineering methods are likely necessary to form AF tissue constituents.^12^ Moreover, AF tissue maintenance and repair depends on lower cell numbers than other cartilage tissues. The cell density of articular cartilage is estimated at 14000‐15000 cells/mm^3^;^34^ whereas, it is as low as 6000 cells/mm^3^ on average in the total IVD,^34^ with reports as low as 3000‐4000 cells/mm^3^ for the AF.^41^ In addition, the AF is largely an avascular tissue, therefore limiting its nutrient supply. Another consideration is the continuous and relatively large forces applied on the AF from all directions, further impeding tissue repair.^42^ The main obstacle to AF regeneration is enabling cell engraftment into the AF under these stringent conditions.^39^ In addition, unlike the NP, the AF is not tightly enclosed by tissue structures that could retain an implant, therefore designing novel methods by which cells or tissues could adhere and engraft become even more important.

In recent years, functional cellular markers for AF cells, such as CD146^23^ and Mohawk^43^ have been identified, offering opportunities for AF cell repair. Despite the many studies that have been performed on transplanting cells to AF, no definitive transplantation methods have been established.^44^ Moreover, in vivo AF regeneration studies have resulted in mixed but suboptimal outcomes.^44^ Decellularized AF grafts applied in rat annuluotomy‐mediated AF‐defect rat model for AF repair resulted in large scar tissue formation.^45^ On the other hand, application of crosslinked collagen constructs applied in a rat needle‐puncture model, showed strongly enhanced IVD features compared to nontreated discs, but still reported atypical AF tissue organization.^46^, ^47^ Noteworthy, however, is that none of these studies applied engraftment of cells within their scaffold product. Work by Sato et al,^16^ applying a rabbit puncture and nucleotomy model revealed that AF repair could significantly be enhanced by engrafting their specialized atelocollagen graft with AF cells, compared to nonseeded atelocollagen products. Disc height and extracellular matrix (ECM) compositions were improved, but their study could not establish native AF architectural organization. Li et al^48^ compared dexamethasone treatment to dexamethasone and bone marrow‐derived mesenchymal cell treatment for AF defect rabbit model created by AF puncture and nucleotomy. Their results similarly indicated improved DHI maintenance and enhanced type II collagen content for the cellular treated group. Overall, these small animal model studies seem to suggest beneficial outcomes for cell treated defect. Our work concurs with these findings, presenting overall improvement in DHI and ECM maintenance, but not to healthy conditions. Overall, these observations suggest that restoring AF architecture and repair of the inner AF remains the main hurdle for AF repair.^44^

The reported cell therapy studies often apply biomaterials and scaffolds to localize cells and seal the defect.^44^ However, biomaterials require extensive preparation and thus limit clinical applicability. Cell sheets are easy to handle as the cells are not dispersed, and a scaffold‐free cell sheet can easily be obtained by simply subjected the culture‐ware to a different temperature. This offers promising translational and commercialization potential, as no (commonly) expensive enzymes or complex cell manipulation procedures are required.^13^, ^14^, ^49^, ^50^

Previous investigations on chondrocytes revealed a strong increase in both SOX9 or COL2 expression in layered chondrocyte sheets compared to a standard monolayer culture.^22^ Not only was chondrocyte phenotype maintained but the gene expression of cell‐adhesion molecules, such as fibronectin or integrin α10, significantly increased and could be detected throughout chondrocyte sheets contributing to the high tissue‐adhesive properties of the constructs.^22^, ^51^ Moreover, investigators found that it is possible to maintain the ECM deposited by the cells while harvesting and transplanting the cell sheets.^52^ As such, application of chondrocyte cell‐sheets in articular cartilage defects of rat knee joints did not require any additional suturing to retain the implants.^53^ Moreover, the implant was reported to be preserved in the transplanted area for >21 months.^53^ High engraftment rates were reported for the myocardium and cornea.^18^, ^54^ In this study, we similarly found the ability of AF cell sheets to attach to the defected area and stay in place without any additional attachment. Moreover, transplanted cell invasion from the cell sheet into AF tissue could be confirmed. The cell‐sheet treated group also showed maintenance of IVD characteristics, such as proteoglycan content, AF structure, and disc height, compared to non‐cell sheet recipients. In short, our results suggest a direct effect from the cell sheet implant in limiting disc degeneration and that the AF cells from the sheet are able to migrate into the AF and contribute to overall IVD repair. In the future, AF cell sheets might be applied in combination with NP cell therapy,^55^, ^56^, ^57^ to further curtail or even reverse IVD degeneration.^10^, ^58^

Although our study provides evidence to the benefits of cell sheet technology for IVD repair, the study does have some limitations. One limitation was the lack of mechanical testing, which will be an important assessment for future studies, in particular with regards to scaling up to human‐sized IVDs. Moreover, no direct assessment could be made of the long‐term architectural changes and collagen fiber orientation in the AF provided by the cell sheet implanted cells. Moreover, future large animal studies will be needed to evaluate the ability of cell sheet implants for limiting disc (re)herniation. Finally, we at this time, have not examined the cell sheet product features or the AF cells applied prior to transplantation. Thus, future studies are required to further analyze and potentially optimize the cell sheet cultures and cell sheet‐grafts potency. Nevertheless, cell sheet technology offers a scaffold‐less transplantation method that provides easy cell handling and cell transplantation that maintains in vitro produced ECM. This approach offers a more accessible and quicker procedure for cell transplantation, implant adherence, and cell engraftment into the challenging AF tissue. The AF demonstrated better morphological maintenance by AF cell sheet treatment in the rat tail AF injury model and could arrest the progression of disc degeneration. In conclusion, cell sheet technology appears to be an effective and safe tool for AF defect repair and as a treatment for IVD degeneration.

## AUTHOR CONTRIBUTIONS

All authors were involved in the study. T.N. was the main researcher involved in surgical procedures and practical data collection. T.N. and J.S. were involved in data analysis, including histological scoring. J.S. and T.N. were involved in drafting of the manuscript. D.S., M.S., and M.W. designed, supervised, and provided funding to the study. All authors have read and approved the manuscript prior to publication.

## CONFLICT OF INTEREST

All included authors declare that they do not have a conflict of interest.

## Data Availability

The data that support the findings of this study are available from the corresponding author (Sakai. D) upon reasonable request.
